# TRENDS IN OSTEOPOROSIS DRUG USE AMONG MEDICARE BENEFICIARIES WITH & WITHOUT ALZHEIMER’S DISEASE/RELATED DEMENTIAS

**DOI:** 10.1093/geroni/igac059.2783

**Published:** 2022-12-20

**Authors:** Peyton Armstrong, Yong-Fang Kuo, Jordan Westra, Mukaila Raji

**Affiliations:** UTMB, Galveston, Texas, United States; University of Texas Medical Branch at Galveston, Galveston, Texas, United States; UTMB, Galveston, Texas, United States; University of Texas Medical Branch at Galveston, Galveston, Texas, United States

## Abstract

**Background:**

Osteoporotic fractures are a leading cause of disability and premature death in the elderly. Patients with Alzheimer’s and related dementia (ADRD) have high rates of osteoporosis (OP) and substantial risk of osteoporotic fractures. Yet research is sparse on trends and predictors of OP medication use in ADRD.

**Methods:**

Medicare beneficiaries with OP aged ≥67 years with Medicare parts A/B/D without HMO from 2016–2018. Outcome was receipt of OP medications in 2018. A multivariable logistic regression assessed association between ADRD and OP drug prescribing, adjusted for age, sex, race, region, Medicare entitlement, dual Medicaid eligibility, chronic conditions, number of provider visits/hospitalizations, and nursing home (NH) resident status. Age/ADRD and NH residency/ADRD interactions were tested.

**Results:**

Sample consisted of 47,871 people with OP and ADRD and 201,840 with OP without ADRD. OP drug use was 38.6% in ADRD patients vs. 52.7% in non-ADRD. After adjustment for demographics, chronic conditions, previous hospitalizations/physician visits, the OR for OP drug in ADRD vs. Non-ADRD was 0.85 (95% CI: 0.83–0.87). NH residents had lower odds for OP medication (OR: 0.61, 95% CI: 0.58–0.64). There were significant interactions between ADRD/age and between ADRD/NH residency. The OR for OP drug use associated with ADRD was 0.88 (95% CI: 0.86–0.90) among community-dwelling elders and 0.66 (95% CI: 0.64–0.69) among NH residents. Conclusions: ADRD patients received OP drugs at lower rates than non-ADRD counterparts. More research is needed on prescribing or deprescribing OP drugs in context of ADRD severity, patient preferences, remaining life expectancy and time-to-benefit from OP drugs.

